# MGRN1 as a Phenotypic Determinant of Human Melanoma Cells and a Potential Biomarker

**DOI:** 10.3390/life12081118

**Published:** 2022-07-26

**Authors:** Marta Abrisqueta, Sonia Cerdido, José Sánchez-Beltrán, Idoya Martínez-Vicente, Cecilia Herraiz, Ana Lambertos, Conchi Olivares, Arrate Sevilla, Santos Alonso, María Dolores Boyano, José Carlos García-Borrón, Celia Jiménez-Cervantes

**Affiliations:** 1 Department of Biochemistry, Molecular Biology and Immunology, School of Medicine, University of Murcia, LAIB Building, Room 1.53, Campus de Ciencias de la Salud, Carretera Buenavista s/n, 30120 Murcia, Spain; marta.ag@um.es (M.A.); sonia.cerdidoo@um.es (S.C.); jose.sanchezb@um.es (J.S.-B.); idoyamaria.martinez@um.es (I.M.-V.); ceciliahs@um.es (C.H.); ana.lambertos@um.es (A.L.); mcolisan@um.es (C.O.); gborron@um.es (J.C.G.-B.); 2 Biomedical Research Institute of Murcia (Instituto Murciano de Investigación Biosanitaria, IMIB), 30120 Murcia, Spain; 3 Department of Cell Biology and Histology, Faculty of Medicine and Nursing, University of Basque Country UPV/EHU, 48940 Leioa, Spain; arrate.sevilla@ehu.eus (A.S.); lola.boyano@ehu.eus (M.D.B.); 4 Department of Genetics, Physical Anthropology and Animal Physiology, Faculty of Science and Technology, UPV/EHU, University of Basque Country UPV/EHU, 48940 Leioa, Spain; santos.alonso@ehu.eus; 5 Biocruces Bizkaia Health Research Institute, 48903 Barakaldo, Spain

**Keywords:** Mahogunin Ring Finger 1 (MGRN1), melanocytes, melanoma, DNA damage, biomarker

## Abstract

Mahogunin Ring Finger 1 (MGRN1), a ubiquitin ligase expressed in melanocytes, interacts with the α melanocyte-stimulating hormone receptor, a well-known melanoma susceptibility gene. Previous studies showed that MGRN1 modulates the phenotype of mouse melanocytes and melanoma cells, with effects on pigmentation, shape, and motility. Moreover, MGRN1 knockdown augmented the burden of DNA breaks in mouse cells, indicating that loss of MGRN1 promoted genomic instability. However, data concerning the roles of MGRN1 in human melanoma cells remain scarce. We analyzed MGRN1 knockdown in human melanoma cells. Transient MGRN1 depletion with siRNA or permanent knockdown in human melanoma cells by CRISPR/Cas9 caused an apparently MITF-independent switch to a more dendritic phenotype. Lack of MGRN1 also increased the fraction of human cells in the S phase of the cell cycle and the burden of DNA breaks but did not significantly impair proliferation. Moreover, in silico analysis of publicly available melanoma datasets and estimation of MGRN1 in a cohort of clinical specimens provided preliminary evidence that MGRN1 expression is higher in human melanomas than in normal skin or nevi and pointed to an inverse correlation of MGRN1 expression in human melanoma with patient survival, thus suggesting potential use of MGRN1 as a melanoma biomarker.

## 1. Introduction

With millions of new cases diagnosed each year, skin cancer represents the world’s most common cancer, with the highest rates observed in fair-skinned populations [1]. Melanoma is the deadliest type of skin cancer since it accounts for only 4% of all cases but is responsible for 80% of skin cancer-related deaths. Exposure to solar ultraviolet radiation (UVR) is the main etiological factor for melanoma in fair-skinned populations due to its DNA-damaging effect [2,3,4]. The individual sensitivity to UVR-induced damage is highly variable and strongly influenced by a well-established skin cancer susceptibility gene: *MC1R.* The *MC1R* gene encodes a G protein-coupled receptor (GPCR) expressed in melanocytes that binds to several melanocortin peptides, including α-melanocyte-stimulating hormone (αMSH) [5]. αMSH is a peptide hormone secreted in a TP53-dependent manner by keratinocytes upon exposure to UVR [6]. Activation of MC1R by αMSH triggers a potent, complex, and multifaceted photoprotective program still incompletely characterized, leading to stimulation of synthesis of photoprotective melanin pigments as well as induction of antioxidant enzymes and DNA repair pathways [5,7]. However, the *MC1R* gene is highly polymorphic, and many *MC1R* variants are associated with a phenotype known as RHC (for **r**ed **h**air **c**olor) consisting of light skin, blond or red hair, inability to tan, and propensity to sunburn. Importantly, extensive genetic analyses of large cohorts have estimated a 60% increased risk of melanoma for carriers of any *MC1R* variant [8], with an additive effect for multiple variants.

It has been demonstrated that the melanoma-associated RHC alleles encode proteins with diminished functional coupling to the cAMP pathway [9,10,11,12,13,14,15,16]. Therefore, it can be hypothesized that deregulation of genes/proteins controlling MC1R signaling through this pathway should also modulate the risk of melanoma and the phenotype of melanoma cells. We have shown that several intracellular proteins that interact physically with MC1R are able to inhibit its αMSH-dependent activation of the cAMP pathway (reviewed in [17]). The most notable is the E3 ubiquitin ligase Mahogunin Ring Finger-1 (MGRN1) [18,19]. The human and mouse *MGRN1* genes are highly homologous. They yield four different alternative splicing isoforms that share exon 10 encoding for a RING Finger domain characteristic of E3 ubiquitin ligases [19,20,21], catalyzing the conjugation of ubiquitin units to target proteins. Several laboratories have shown that MGRN1 displays E3 ligase activity and ubiquitinates several protein substrates [22,23,24,25]. Moreover, all MGRN1 isoforms interact with MC1R to inhibit signaling to cAMP, most likely by competitive displacement of the Gs protein ultimately responsible for adenylyl cyclase activation [19]. 

Since both the RHC mutations of MC1R and the MGRN1–MC1R interaction decrease MC1R-dependent activation of the cAMP pathway, we hypothesized that the level of expression and/or the activity of MGRN1 may still have poorly characterized effects on key properties of melanocytes and melanoma cells. In agreement with this hypothesis, we showed previously that MGRN1 is a key regulator of the phenotype of normal mouse melanocytes and melanoma cells, with marked effects on pigmentation, shape, and motility [26,27]. Moreover, the knockdown of MGRN1 augmented the burden of DNA strand breaks in mouse melanoma cells, thus indicating that loss of MGRN1 led to genomic instability [27]. 

So far, the biological roles of MGRN1 have been most often investigated in cultured mouse cells or laboratory mice, and the data concerning the biological roles of MGRN1 in human melanocytes and melanoma cells are still scarce. Therefore, the potential roles of MGRN1 in melanomagenesis, on one hand, and in the clinical course of the disease, on the other, remain largely unexplored, thus preventing the exploitation of this protein as a potentially useful therapeutic target and/or melanoma biomarker. We present here a study of the effects of MGRN1 knockdown in human melanoma cells. We show that MGRN1-null cells display a more differentiated phenotype and a higher burden of DNA strand breaks, consistent with previous studies performed with murine cell lines. Moreover, we present preliminary evidence suggesting that MGRN1 expression is higher in human melanomas than in normal skin or nevi and pointing to an inverse correlation of MGRN1 expression in human melanoma with patient survival.

## 2. Materials and Methods

### 2.1. Ethics Statement

The study protocol conformed to the tenets of the Declaration of Helsinki (64th WMA General Assembly, Fortaleza, Brazil, October 2013) and was approved by the Euskadi Ethics Committee (PI+CES BIOEF 2020–17). All patients (including those with nevi) gave written informed consent to participate in the study and to use their biopsies as material for research.

### 2.2. Patients and Samples

The study of human samples focused on specimens from patients with histologically confirmed malignant melanoma, previously described by Sevilla et al. [28]. Patients were untreated, other than primary surgery, and disease stages were classified according to the AJCC (American Joint Committee on Cancer). A group of nevi was also included in the study.

### 2.3. Reagents

Laboratory reagents and protease or phosphatase inhibitors were from Sigma (St. Louis, MO, USA), Calbiochem (Darmstadt, Germany), Merck (Darmstadt, Germany), or Prolabo (Barcelona, Spain), unless specified otherwise. Lipofectamine 2000 and Opti-MEM I were from Gibco (Gaithersburg, MD, USA). Reagents for SDS-PAGE and Western blot were from Bio-Rad (Richmond, CA, USA).

### 2.4. Cell Culture and Generation of MGRN1-KO Cells

**Figure 1:**
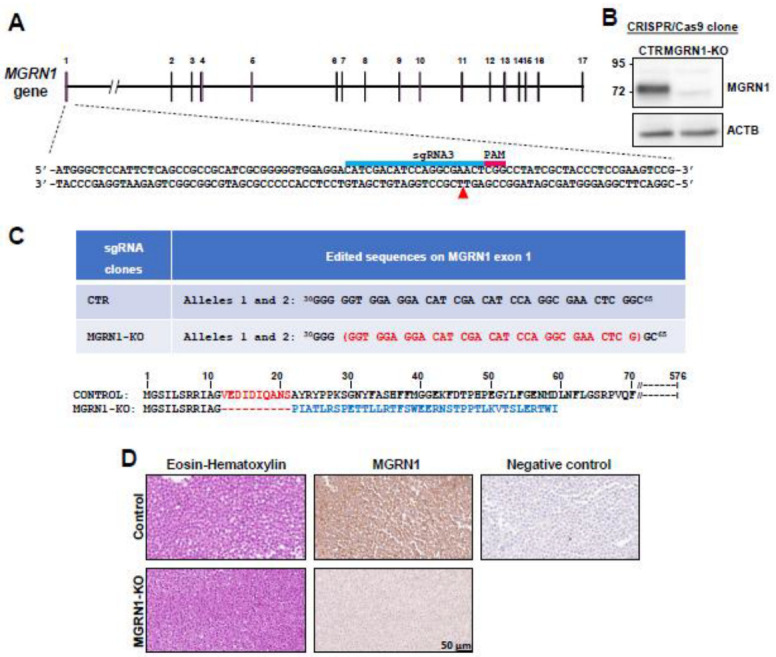
CRISPR/Cas9-mediated knockdown of MGRN1 in HBL human melanoma cells. (**A**) Schematic representation of the *MGRN1* gene highlighting the sequence targeted by sgRNA3 in exon 1 (blue bar). This 20 mer oligonucleotide was designed to bind the DNA target directly upstream of a 5′-NGG adjacent motif (PAM, red bar). (**B**) Representative immunoblot of MGRN1 in control and MGRN1-KO 3.7 clonal cells. Cell-free detergent-solubilized extracts were electrophoresed in 10% SDS-PAGE gels and immunoblotted for MGRN1 and actin B (ACTB) as a control for comparable loading. Note the different electrophoretic mobility of the MGRN1 band in the control lane and the faint non-specific band in the extract of MGRN1-KO cells. (**C**) Top: edited sequence in exon 1 of MGRN1 in MGRN1-KO-3.7 cells, compared with the consensus sequence in control (CTR) HBL cells (nucleotides 30 to 65). Deleted nucleotides are shown in red, between brackets. Bottom: amino acid sequence of control and the resulting truncated MGRN1 protein in MGRN1-KO-3.7 cells. (**D**) Homogeneity of clonal MGRN1-KO cell cultures. Control and MGRN1-KO cells were eosin–hematoxylin-stained or immunostained for MGRN1, as indicated. Note the lack of significant staining for MGRN1-KO cells, indicating the homogeneity of the clonal culture. Negative control stands for samples of control MGRN1-expressing cells treated with a secondary antibody in the absence of anti-MGRN1.

Cell culture reagents were from Gibco (Gaithersburg, MD, USA). HBL cells (LOCE-MM1 cells) were kindly provided by Prof G Ghanem, LOCE-Institut J. Bordet, Université Libre de Bruxelles, Belgium. They were grown in DMEM-GlutaMAX with 10% fetal bovine serum, 100 U/mL penicillin, and 100 μg/mL streptomycin sulfate. To knock down MGRN1 in these cells, we used target sequences for CRISPR-RNA from Dharmacon specified in Figure 1. Efficiencies and potential off-targets were estimated with *Breaking-Cas* (http://bioinfogp.cnb.csic.es/tools/breakingcas) [29]. Cells were co-transfected with 2.5 µg Cas9 Nuclease Expression plasmid with puromycin selection marker, 50 nM trans-activating CRISPR RNA (tracrRNA), 50 nM CRISPR-RNAs, and 6 µg/mL DharmaFECT Duo Transfection Reagent (all from Dharmacon). For negative control cells, we transfected 50 nM crRNA Non-targeting Control #1. After incubation at 37 °C for 72 h, puromycin-resistant clonal cells were screened for MGRN1 knockdown by Western blot, performed as described [9,27]. Confirmation of selected clones was performed by automated sequencing after PCR amplification of a 700 bp fragment on exon 1 using genomic DNA as a template.

### 2.5. siRNA-Mediated Repression of MGRN1 in Cultured Cells

HBL cells were transfected with a stoichiometric mixture of human MGRN1 siRNA oligonucleotides siGENOME 1–4 or control non-targeting siRNA, at a final concentration of 30 nM, using OptiMEM and 5 µL/well DharmaFECT 4 as transfection reagent. The sequence of these siRNAs is specified in Table 1. Unless otherwise specified, the cells were kept in the continuous presence of siRNA for 72 h before analysis.

### 2.6. Cell Proliferation and Cell Cycle Assays

Cells were seeded and manually counted using a hemocytometer at different time points from 24 to 96 h. Normalized cell number values were plotted using GraphPad Prism (GraphPad Software, San Diego, CA, USA, www.graphpad.com) and doubling times were calculated by nonlinear regression using an exponential growth equation. Two other methods were also employed. In both cases, equal numbers of control and MGRN1-KO cells were seeded on 96-well plates and grown as described above for 48 h. Then, DNA synthesis was estimated by incubating cultures in exponential growth with 20 µM BrdU for 1 h in complete medium. Next, cells were washed with PBS and labeled with a mouse anti-BrdU antibody followed by an Alexa 488-labeled anti-mouse secondary antibody and measurement of fluorescence intensity. To perform MTT metabolic activity assays, cells were washed with phenol red-free medium and incubated for 2–3 h with MTT (final concentration 0.2 mg/mL in fresh medium without phenol red). The medium was gently aspirated, the cells were solubilized in 100 µL DMSO with gentle shaking, and the absorbance at 562 nm was measured in a microplate reader. For cell cycle analysis, cells were fixed in ethanol 70% in PBS, pelleted, and resuspended in PBS containing 100 µg/mL RNase A and 40 µg/mL propidium iodide (PI), and analyzed in an FACScanto cytometer (BD Biosciences). 

### 2.7. Confocal Fluorescence Microscopy and Analysis of H2AX Phosphorylation 

Cells were fixed in 4% formaldehyde, permeabilized, and blocked in BSA. DNA was stained with DAPI. For γ-H2AX staining, cells were processed as described [7]. Images were taken with an SP8 Leica laser scanning confocal microscope and software (Leica Microsystems GmbH, Wetzlar, Germany) with HCXPL APO CS 40× or 63× objective lenses. Nuclear γ-H2AX fluorescence signal was quantified by calculating the pixel intensity in single cell nuclei relative to the nucleus area. At least 200 randomly selected cells per condition were quantified using ImageJ.

### 2.8. Comet Assays

The alkaline comet assay was performed as previously described [7,27] according to the manufacturer’s protocol (Trevigen, Gaithersburg, MD, USA).

### 2.9. Analysis of Gene Expression from Human Databases

TCGA (https://portal.gdc.cancer.gov/) and GEPIA (http://gepia.cancer-pku.cn/) databases were employed to compare MGRN1 expression levels and to obtain survival curves from human melanoma samples normalized data.

### 2.10. Real-Time RT-PCR and Digital PCR (dPCR)

Real-time quantitative PCR was performed to assess the levels of expression of MGRN1 in cultured cells treated with control or MGRN1-directed siRNA. To this end, total RNA was extracted with the commercial kit RNeasy Mini Kit (QIAGEN, Hilden, Germany). Reverse transcription of RNA (1 μg) was performed with the SuperScript II First-Strand Synthesis kit (Invitrogen Life technologies™, Carlsbad, CA, USA) as per the manufacturer’s instructions. qRT-PCR was performed using the Power SYBR Green PCR Master Mix (Applied Biosystem, Foster City, CA, USA) on an ABI 7500 Fast Real-Time PCR System. Actin B (ACTB) mRNA was used as the reference gene for normalization. Primer sequences are specified in Table 2.

To compare the expression of MGRN1 in paraffin-embedded clinical samples, we used digital PCR (dPCR). Unless otherwise specified, reagents and equipment for dPCR were from Applied Biosystems [28]. RNA isolated from 49 nevi and 33 melanomas biopsies was first converted to cDNA using the Kit Maxima First Strand cDNA Synthesis Kit (ThermoFisher, Waltham, MA, USA), following the manufacturer’s instructions. To detect gene expression differences smaller than twofold, dPCR was performed on the QuantStudio™ 3D Digital PCR System. First, we mixed cDNA with QuantStudio™ 3D Digital PCR Master Mix V2 and Taqman Gene Expression Assays (Hs01005100_m1 for MGRN1 and Hs02758991_g1 for GAPDH, both from Applied Biosystems). The resulting dPCR reaction mix was loaded onto a QuantStudio™ 3D Digital PCR Chip v2, containing 20,000 independent reaction wells. To detect the presence or absence of DNA molecules of interest in each independent reaction chamber, PCR was performed using the ProFlex™ 2× Flat PCR System and read using the QuantStudio™ 3D Digital PCR Instrument. The instrument determines the location and intensity of the fluorescent signals in each image, the dye associated with each fluorescent signal, and the significance of the signal. Finally, data were analyzed using the QuantStudio™ 3D AnalysisSuite™ Software, which generates copies/μL for each sample based on copies/reaction generated and the dilution factor of the sample. To analyze gene expression differences between samples, we used dPCR quantification of GAPDH as endogenous control. Results were calculated as follows:

Relative MGRN1 expression (%) = (MGRN1 copies/μL)/(GAPDH copies/μL) × 100(1)

### 2.11. Statistical Analysis

All analyses were carried out using GraphPad Prism. For comparison of control and MGRN1-KO cells, unpaired two-tailed Student’s t-tests were performed, and data are given as mean ± standard error of the mean (sem) unless indicated otherwise. Kaplan–Meier survival curves were compared using a log-rank test. *p* values of less than 0.05 were considered statistically significant. * Indicates *p* < 0.05, ** *p* < 0.01, *** *p* < 0.001, and **** *p* < 0.0001.

## 3. Results

In previous studies, we found that MGRN1-KO B16 mouse melanoma cells displayed a more differentiated phenotype with increased pigmentation, higher adhesion to collagen I, and low motility in 2D and 3D assays, compared to control cells. Moreover, when injected into the tail vein, MGRN1-KO B16 cells colonized the lungs of mice with much lower efficiency than control cells [26,27]. Therefore, it was of interest to analyze whether knockdown of MGRN1 in human melanoma cells caused a similar phenotypic switch. To this end, we abolished MGRN1 expression in HBL human melanoma cells by CRISPR/Cas9. HBL cells are wildtype (WT) for the *NRAS*, *BRAF,* and *TP53* genes and they have been widely used to study MC1R signaling [7,9,10,11,19]. For MGRN1 knockdown, we designed a guide RNA targeting exon 1 (sgRNA3, Figure 1A) to alter the protein sequence near its N-terminus, thus avoiding the conservation of potentially functional domains. We selected several MGRN1-KO clones for estimation of MGRN1 protein levels and *MGRN1* sequencing. One of these clones (clone MGRN1-KO-3.7, from now on MGRN1-KO for simplicity) was considered for further study since Western blot analysis confirmed the absence of MGRN1 (Figure 1B) and *MGRN1* sequencing revealed a deletion of 31 nucleotides resulting in loss of 10 amino acids after Glycine 11 followed by a change in the reading frame and appearance of a premature stop codon, thus leading to early truncation of the protein and ensuring complete MGRN1 loss of function (Figure 1C). Finally, to ascertain the homogeneity of our MGRN1-KO clonal cells and to exclude cross-contamination of control and MGRN1-null cultures, we stained paraffin-embedded, formaldehyde-fixed cellular pellets with eosin–hematoxylin and with an anti-MGRN1 antibody. As shown in Figure 1D, both control and MGRN1-KO cell cultures were homogeneous, with a strong positive MGRN1 immunostaining for control cells and a background signal for the negative control and MGRN1-KO cells. Moreover, MGRN1-KO cultures did not contain MGRN1-positive cells.

**Figure 2:**
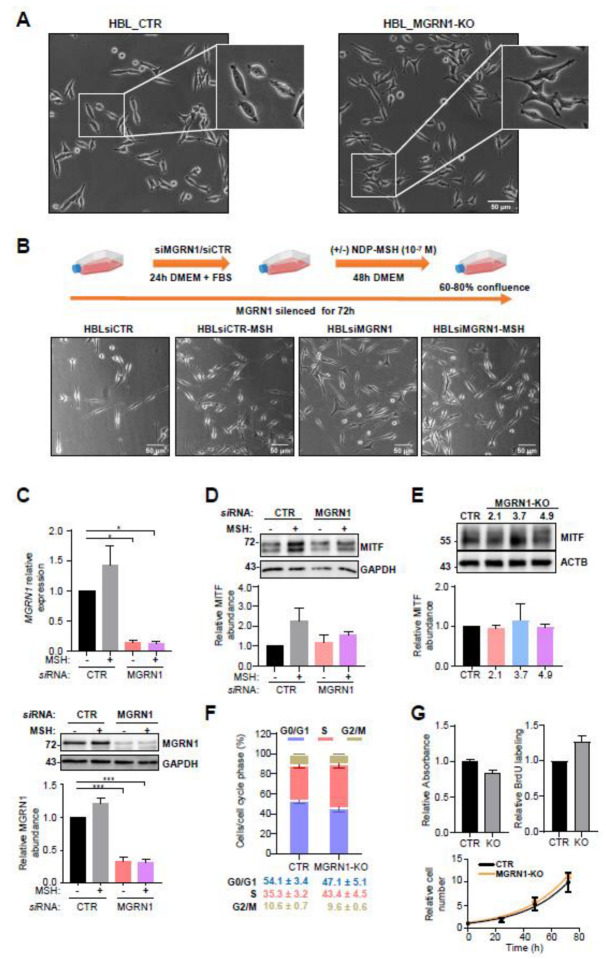
Normal growth of MGRN1-null human melanoma cells. (**A**) Phase-contrast images of control (HBL-CTR) and MGRN1-KO cells (HBL MGRN1-KO) in 2D cultures. The smaller images on the top right corners show a magnification of a representative region of the micrograph. Scale bar, 50 μm. (**B**) Changes in the morphology of HBL cells depleted of MGRN1 by siRNA transfection and stimulated with NDP-MSH, as specified in the upper scheme depicting the experimental design. Representative phase-contrast micrographs are shown below. (**C**) Efficient repression of MGRN1 expression by the siRNA transfection employed above. The upper graph represents the levels of MGRN1 mRNA as estimated by real-time RT-PCR, normalized to the expression in cells treated with control, non-targeting siRNA. ACTB mRNA was used as a loading control. Further details concerning primer sequences are provided under Material and Methods. A representative Western blot of detergent-solubilized cell extracts, stained for MGRN1 and GAPDH as loading control, is shown below, along with the quantification of 3 independent blots. (**D**) Western blot analysis of MITF expression in cells treated as in panel B, immunostained for MITF and GAPDH (as loading control). A representative blot out of 3 independent experiments and the quantification of the fold change of the normalized MITF signal relative to control unstimulated cells is shown below. (**E**) Comparable expression of MITF in control HBL cells and MGRN1-KO clones obtained by permanent knockdown of MGRN1. Detergent-solubilized, cell-free extracts were analyzed for MGRN1 by Western blot. A representative blot out of 3 independent experiments and the corresponding quantification are shown. The numbers on top of the MGRN1-KO lanes refer to the individual clone analyzed. GAPDH was used as a loading control. The numbers below these lanes correspond to the normalized MITF signal relative to the control lane (mean of 2 independent experiments). (**F**) Cell cycle progression in human melanoma cells lacking MGRN1. Control HBL cells and MGRN1-KO cells (clone 3.7) were stained with propidium iodide and analyzed in an FACScanto cytometer. The graph shows the percentage of cells in the G0/G1, S, and G2/M phases (mean ± sem, n = 5 for control and n = 2 for MGRN1-KO cells). (**G**) Comparable proliferation rates of control and MGRN1-KO cells. The proliferation of control and MGRN1-KO cells was compared with 3 independent methods: metabolic activity measured with MTT (**left**), metabolic labeling of DNA with BrdU (**right**), and counting viable cells over 72 h (graph on the bottom). In this last case, doubling times of roughly 23 h were obtained upon nonlinear regression, without statistically significant differences.

Consistent with previous observations in cultured mouse melanocytes, MGRN1-KO human cells grown on plastic bottles showed a more differentiated phenotype as evidenced by a higher fraction of dendritic cells. Moreover, these cells had apparently longer dendritic processes (Figure 2A). To rule out potential artifacts due to the CRISPR/Cas9 treatment or to the selection of an individual clone, we wished to confirm this modulation of the cellular phenotype by transient, siRNA-mediated repression of MGRN1 in HBL cells.

As schematized in Figure 2B, HBL cells seeded on plastic flasks were treated with control or MGRN1-specific siRNA in a serum-free medium, then challenged with the MC1R agonist NDP-MSH, used as a positive control for cell morphology changes, at a saturating final concentration of 10^−7^ M for 48 h. Downregulation of MGRN1 with a specific siRNA or treatment with NDP-MSH increased the dendricity of HBL cells (Figure 2B), resulting in a phenotype comparable to MGRN1-KO cells. Efficient depletion of MGRN1 by the siRNA treatment was confirmed by measurement of MGRN1 mRNA and protein levels (Figure 2C). Accordingly, the effects of acute, short-term siRNA-mediated downregulation of MGRN1, on one hand, and complete permanent knockdown by CRISPR/Cas9, on the other, were similar. In mouse melanocytes and melanoma cells, a comparable phenotypic change was observed upon repression of MGRN1 [27]. This change was independent of the master regulator of melanocyte proliferation and differentiation Microphthalmia Transcription Factor (MITF) [30,31]. Therefore, it was of interest to analyze possible changes in MITF expression upon downregulation of MGRN1 in HBL cells. As shown in Figure 2D, Western blot analysis indicated that depletion of MGRN1 did not significantly augment the levels of MITF. As a further internal control for this experiment, the effects of NDP-MSH on MITF levels were also analyzed (Figure 2D). As expected, NDP-MSH treatment augmented MITF levels in control HBL cells. Of note, MITF expression was similar in control cells and cells depleted of MGRN1 by siRNA treatment. In addition, we also compared by Western blot MITF expression in control HBL cells and several independent clones of MGRN1-KO cells. Again, no significant differences in MITF levels were observed for cells expressing or not MGRN1, including those of the 3.7 clone used throughout this work (Figure 2E). In summary, transient depletion or permanent ablation of MGRN1 in human melanoma cells caused an apparently MITF-independent switch to a more dendritic phenotype, consistent with previous observations in mouse melanocytes. 

Concerning proliferation, the cell cycle profiles of control and MGRN1-KO cells were comparable, although a trend towards a higher fraction of cells in the S phase was observed (Figure 2F), in agreement with our previous findings in MGRN1-deficient mouse melanocytic cells [27]. The rate of proliferation was measured by three independent methods, with somewhat different results. BrdU incorporation was used to label newly synthesized DNA. Slightly higher incorporation of the label was observed for MGRN1-KO cells (Figure 2G, bar graph on the left). However, when the metabolic activity of cell cultures was measured using the MTT assay, MGRN1-KO cells showed a small but significant decrease (Figure 2G, bar graph in the middle). Finally, direct counting of viable cells yielded similar doubling times for control and MGRN1-KO cells (Figure 2G, graph on the right). Taken together, these data showed that the small perturbation of cell cycle formation was not sufficient to significantly impair the rate of proliferation of MGRN1-null cells.

**Figure 3:**
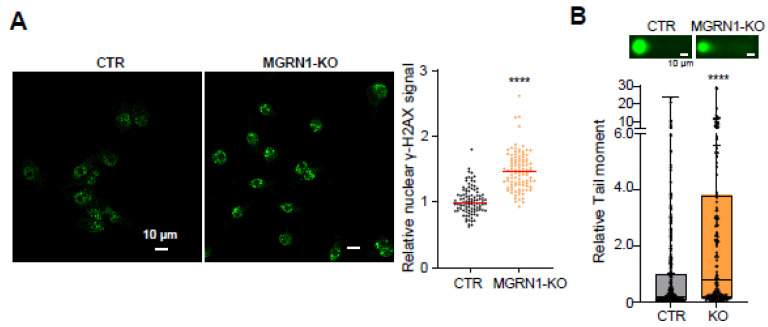
Increased DNA damage in MGRN1-null human melanoma cells. (**A**) Representative images of control (CTR) and MGRN1-KO cells immunostained for γ-H2AX (**left**) and quantification of γ-H2AX staining intensity (**right**). (**B**) Alkaline comet assay was performed to detect DNA breaks in control (CTR) and MGRN1-KO cells. Representative images of comets are shown. The graph shows the median of relative tail moments measured using CASPLAB software.

We next analyzed the presence of DNA strand breaks, since we previously reported an increased burden of this type of lesion in MGRN1-deprived mouse melanocytes and melanoma cells. To this end, we compared the levels of the phosphorylated form of histone H2AX (γ-H2AX) in control and MGRN1-KO human melanoma cells using an immunocytochemical technique. H2AX is quickly phosphorylated in response to DNA damage, forming nuclear foci near double-strand breaks (DSBs) to foster recognition and repair of these lesions [32]. The presence of γ-H2AX foci is therefore a useful marker of DNA DSBs. As shown in Figure 3A, MGRN1-KO cells presented significantly higher levels of γ-H2AX labeling compared with control cells, pointing to a higher burden of DSBs in human melanoma cells lacking MGRN1. To confirm this finding, we used the comet assay to quantify DNA damage. Quantitative tail moment measurements in alkaline comet assays confirmed a significantly higher abundance of DNA breaks in MGRN1-KO cells (Figure 3B), consistent with γ-H2AX labeling.

**Figure 4:**
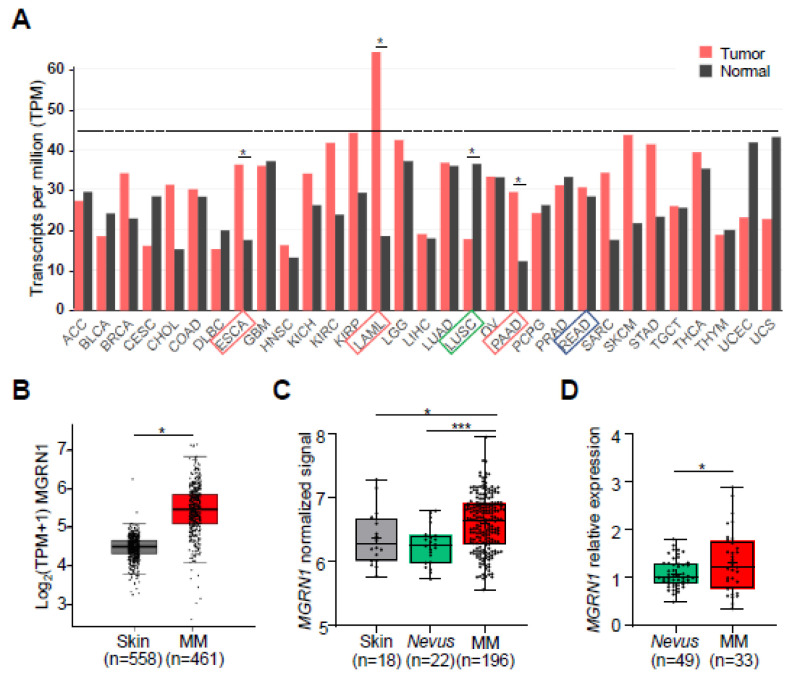
Expression of MGRN1 in melanomas, nevi, and normal skin. (**A**) Analysis of MGRN1 expression across different tumor types (TCGA dataset) and normal tissues (GTEx dataset), available in the GEPIA database. Median expression is represented by the height of the bar. Tumor abbreviations in X axis highlighted with red boxes indicate a statistically significant higher expression of MGRN1 in tumors than in paired normal samples. Green boxes represent lower expression of MGRN1 in tumor. SKCM stands for skin cutaneous melanoma. ANOVA was used for statistical analysis. (**B**) Representation of the normalized MGRN1 expression levels in normal skin and melanoma samples, available in the GEPIA database. (**C**) Comparison of the normalized MGRN1 expression levels in normal skin, nevi, and melanoma samples, using six independent datasets available at the GEO database. (**D**) Relative expression of MGRN1 in nevi and melanoma samples obtained at the BBHRI. MGRN1 mRNA levels were quantified by dPCR and GAPDH mRNA was used for normalization. About 40 samples per group were analyzed. Graphs show mean ± sem, and one-way ANOVA was used for statistical analysis. * *p* < 0.05. In panels (**B**–**D**), MM stands for melanoma.

Accordingly, it appeared that knockdown of MGRN1 in human melanoma cells promoted a phenotype characterized by higher differentiation and genomic instability, comparable with the one previously described for mouse melanoma cells [27]. It was therefore interesting to test the possibility that the level of MGRN1 expression in human melanoma tumors might have an impact on the course of the disease. To this end, we interrogated public databases for correlations between the expression of MGRN1 and relevant clinical parameters and we also used a collection of melanoma specimens previously obtained at the Biocruces Bizkaia Health Research Institute (BBHRI). This sample cohort has been described and successfully used in previous studies [28,33], and the relevant clinical information on the patients is available. Using the data compiled in the GEPIA database (http://gepia.cancer-pku.cn/), we compared the relative expression of the *MGRN1* gene in different types of tumors from human patients and paired normal tissues (TCGA and GTEx cohorts), as well as in melanoma compared with normal skin. This analysis indicated that cutaneous melanoma (SKCM) is one of the cancers with a higher expression of MGRN1. In fact, only in acute myeloid leukemia (LAML) was MGRN1 expression clearly higher than in melanoma (Figure 4A). 

Interestingly, these data also suggested that the expression of MGRN1 might be higher in melanoma compared with healthy skin (Figure 4B), again pointing to a role for MGRN1 in the malignant phenotype of melanoma cells. Furthermore, the analysis of pooled normalized data from six independent datasets in the Gene Expression Omnibus (GEO) database (https://www.ncbi.nlm.nih.gov/geo/) confirmed a significant enrichment of MGRN1 in melanoma compared with either normal skin or nevi (Figure 4C). We wished to extend this in silico analysis to a collection of nevi and melanoma biopsies. To this end, we compared *MGRN1* mRNA levels across the BBHRI cohort by digital PCR (dPCR), a technique that can accurately detect gene expression differences smaller than twofold. Interestingly, we found a similar trend since this study also pointed to a lower expression in nevi compared with melanoma (Figure 4D) with a statistically significant difference. The data summarized thus far suggested that MGRN1 is overexpressed in melanoma compared with normal skin and has similar effects on the phenotype of human melanoma cells compared with mouse melanoma cells. Therefore, MGRN1 might have an important role in modulating the behavior of human melanomas and may even be a determinant of melanoma prognosis. 

**Figure 5:**
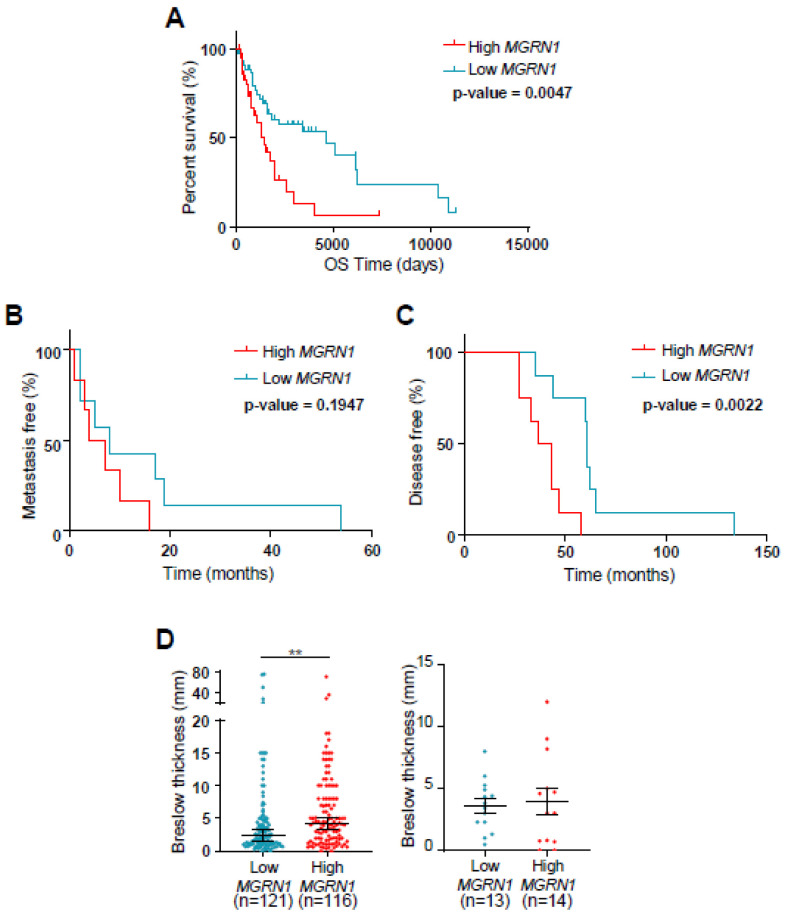
Correlation of lower MGRN1 expression in melanomas with longer patient survival. (**A**) Kaplan–Meier curves for the survival of melanoma patients according to data from the TCGA dataset. Patients were stratified as a function of the highest and lowest 10% levels of MGRN1 in primary tumor specimens. (**B**,**C**) Kaplan–Meier curves show metastasis-free (**B**) and disease-free (**C**) survival for patients of the BBHRI upon resection of primary melanomas. Patients were stratified as a function of the 50% high (blue curves, n = 6 for left graph and n = 8 for right graph) and 50% low (red curves, n = 6 for left graph and n = 8 for right graph) MGRN1 mRNA levels in primary tumor specimens. *p* values are indicated in the graphs. (**D**) Comparison of the Breslow thickness at diagnosis in melanoma samples. Data correspond to the TCGA dataset (**left**) with the 25% higher and lower expression of MGRN1, and to the BBHRI cohort (right) with the 50% higher and lower MGRN1 expression. Graphs show mean ± sem (**right**) or median (**left**), and a t-test was used for statistical analysis.

This possibility was further suggested by our previous in silico analysis of correlations of high and low MGRN1 mRNA levels and clinical data in a melanoma cohort of > 450 melanoma patients (TCGA dataset, https://www.cancer.gov/about-nci/organization/ccg/research/structural-genomics/tcga, also available through xena.ucsc.edu). In this analysis, we defined high- and low-expression tumors as the upper and lower 33% MGRN1-expressing tumors, respectively, and a significant difference was found in the median overall survival between both groups (median survival for patients with low-MGRN1-expressing tumors of 3136 days versus 1832 days for the high MGRN1 group, *p* = 0.0146) [27]. When we used a more restrictive analysis by considering the groups of tumors with the highest and lowest 10% expression of MGRN1, the corresponding Kaplan–Meier curves became even more separated, with a median survival of 1487 and 4648 days, respectively (*p* = 0.0047) (Figure 5A). 

To validate this in silico analysis we measured MGRN1 expression by dPCR in our cohort of melanoma biopsies. Owing to the limited number of samples, in this case we defined the high and low MGRN1 expression groups as the upper and lower 50% MGRN1-expressing samples, respectively. We constructed Kaplan–Meier curves for metastasis-free survival, defined as the time, in months, from the diagnosis of melanoma to the appearance of metastasis in those patients who developed metastasis during the follow-up (Figure 5B). We also analyzed disease-free survival, i.e., the time from diagnosis of melanoma to the end of the study in those patients who did not develop metastasis during the follow-up (Figure 5C). Interestingly, we observed that patients with high-MGRN1-expression tumors had a significantly shorter disease-free period than patients with low MGRN1 expression (*p* = 0.0022). A similar trend was observed for the metastasis-free patients, although in this case the difference did not reach statistical significance, most likely due to the limited number of samples and the fact that samples were divided into groups corresponding to 50% higher or lower expression, rather than in three groups to compare the 33% higher or lower expression samples. The prognostic indicators most commonly used in melanoma are a small set of histopathological factors such as Breslow index, presence or absence of ulceration, level of local invasion, and mitotic rate. We interrogated the TCGA dataset for differences in Breslow thickness between high- and low-MGRN1-expressing samples. We found that high expression of MGRN1 was associated with a higher tumor thickness at diagnosis, with a strong statistical significance (*p* = 0.0012) (Figure 5D). To further analyze this point, we performed a similar analysis with the melanoma samples of the BBHRI cohort. We confirmed a trend towards a higher Breslow index for the high-MGRN1-expression tumors, although in this case the difference was not statistically significant, most likely because of the limited size of the cohort and the different stratification of the samples, as mentioned for Kaplan–Meier analysis. Nevertheless, these findings again suggested a negative correlation between high MGRN1 expression and disease outcome, further underscoring its potential use as a melanoma biomarker.

## 4. Discussion

The *MC1R* gene encoding for the αMSH receptor is a well-characterized melanoma susceptibility gene [5,8]. Several hypomorphic variants associated with increased melanoma risk show a diminished functional coupling to the cAMP signaling pathway, and hence an impaired ability to trigger photoprotective responses (reviewed in [5]). Therefore, it is widely assumed that impaired signaling to the cAMP pathway downstream of MC1R is an important factor in melanomagenesis, particularly in fair-skinned individuals. Accordingly, we hypothesized that negative regulators of MC1R functional coupling may also modulate the susceptibility to melanoma and/or the course of the disease. Several intracellular proteins that interact physically with MC1R to decrease its ability to activate the cAMP cascade have been identified [17]. These include the cytosolic β-arrestins responsible for desensitization of GPCRs [34], the negative regulator of the AKT pathway PTEN [35], and the RING Finger domain-containing E3 ubiquitin ligase Mahogunin Ring Finger-1 (MGRN1) [19]. MGRN1 was identified by positional cloning of mahoganoid (md) [20], a coat color mutation with pleiotropic and complex effects including spongiform neurodegeneration with features of prion diseases [24] and congenital heart defects [25,36]. Mice homozygous for the *md* loss-of-function mutation of the *Mgrn1* gene show a darker coat color similar to mice with gain-of-function mutations at the *Extension* locus coding *Mc1r*. Consistent with this finding, we showed previously that abrogation of MGRN1 expression in mouse melanocytes and B16 mouse melanoma cells by CRISPR/Cas9 led to a more dendritic and differentiated phenotype with higher melanin content [26]. Interestingly, these MGRN1-deficient cells also showed high genomic instability, increased adhesion to collagen, and decreased motility in both wound healing and invasion assays in vitro. Importantly, when equivalent numbers of fluorescently labeled control and MGRN1-KO cells were co-injected into the tail vein of mice, MGRN1-null cells had a markedly lower ability to colonize the lungs of C57BL mice [27]. Therefore, ablation of MGRN1 expression in mouse melanoma cells decreased their metastatic potential.

Here, we have extended these studies to human cells by analyzing several aspects of the phenotype of HBL melanoma cells after CRISPR/Cas9-mediated knockdown of the *MGRN1* gene. Clonal MGRN1-null human melanoma cells were viable and did not show significant alterations in their cell cycle progression and proliferation rates, at least under standard cell culture conditions. However, they displayed a more differentiated phenotype as shown by a more dendritic shape. MGRN1-KO human melanoma cells also displayed an increased genomic instability, with a higher burden of DNA strand breaks. Accordingly, the effects of MGRN1 knockdown on the phenotype of human and mouse melanoma cells were similar. 

Our previous finding that MGRN1-null mouse melanoma cells are less aggressive than control cells expressing normal levels of MGRN1 suggested that MGRN1 might be a useful biomarker in melanoma. However, the possible use of MGRN1 as a prognostic marker for the clinical management of the disease has not yet been investigated. Therefore, we analyzed possible correlations between the level of MGRN1 expression and clinical parameters in human melanoma patients, using a combination of in silico analyses of data compiled in public databases and preliminary studies in a cohort of nevi and melanoma biopsies obtained at the BBHRI. We found that MGRN1 is overexpressed in human melanomas compared with normal skin. Indeed, data compiled in the GEPIA and TCGA databases showed that *MGRN1* expression in surgical melanoma specimens was significantly increased compared to normal skin. In addition, both in silico analysis and direct comparison of *MGRN1* mRNA levels in nevi and melanoma samples from the BBHRI cohort suggested that *MGRN1* gene expression might be higher in melanomas compared with nevi. Although further studies with a larger number of clinical samples are required to increase the statistical significance, these data suggest that the transition from a benign to a premalignant or malignant lesion could be accompanied by an increase in the expression of MGRN1.

Given that the similar phenotypes of MGRN1-null mouse and human melanoma cells and our previous findings that impaired MGRN1 expression in mouse melanoma cells decreased their motility and invasive potential, we hypothesized that low expression of MGRN1 in human melanoma may also result in a less aggressive phenotype. Importantly, Kaplan–Meier analysis of data from TCGA showed that higher expression of MGRN1 in the melanoma samples was associated with shorter survival of patients, and this was further suggested by the direct measurements of MGRN1 expression in the cohort of clinical specimens from the BBHRI reported here. 

Despite recent improvements in the available therapeutic options, the rate of mortality from melanoma remains high largely due to two factors. On one hand, melanoma is one of the tumors with the highest metastatic potential, even when the primary lesion is only a few millimeters in depth [37]. On the other hand, even if treatment of metastatic melanoma has improved in the last decade due to advances in targeted chemotherapy and immunotherapy, objective responses remain most often transient in responders and the rates of non-responders are high [38]. Therefore, it is still required to design new treatment options as well as improved methods to guide the clinical management of the disease. In this respect, hundreds of studies aiming at identifying potential molecular markers that may predict the course of cutaneous melanoma have been reported (reviewed in [39]), but further efforts in this direction are still urgently needed. So far, melanoma-specific antigens (such as Melan-A, or PMEL17 recognized by the HMB45 monoclonal antibody) are widely used for the immunochemical assessment of melanoma specimens, and S100β is considered a marker of relapse or hidden metastases, but its utility is under discussion and no molecular method to improve risk stratification is commonly used in the clinical practice. In this setting, the determination of MGRN1 expression in clinical melanoma specimens might provide useful prognostic information. Work is underway to analyze possible changes in the motility and invasive potential of MGRN1-depleted human melanoma cells, and to further probe the clinical application of the determination of MGRN1 expression levels in melanoma by the analysis of larger cohorts of patients.

## 5. Conclusions

1. Transient MGRN1 depletion or permanent knockdown in human melanoma cells led to a differentiated phenotype by an apparently MITF-independent mechanism.
2. Depletion of MGRN1 altered cell cycle progression and induced genomic instability by promoting the accumulation of DNA strand breaks.
3. In silico analysis of melanoma datasets showed that MGRN1 expression is higher in human melanomas than in normal skin or nevi and that there is a significant correlation between lower MGRN1 expression in human melanoma with longer patient survival. This was confirmed by the estimation of MGRN1 expression in a cohort of nevi and skin melanoma specimens.
4. Therefore, analysis of MGRN1 expression might provide useful clinical information. Accordingly, MGRN1 should be further analyzed as a potential melanoma biomarker.

## Figures and Tables

**Table 1:** Sequence of siRNA oligonucleotides for transient repression of MGRN1 expression.

siRNA	Sequence (5′-3′)
Non-Targeting siRNA (siCTR)	UAAGGCUAUGAAGAGAUAC
siMGRN1 1	CGAAGGAGAUGUGGUGGAA
siMGRN1 2	GAGCACCUUGUCCCUUUA
siMGRN1 3	GAACUCGGCCUAUCGCUAC
siMGRN1 4	AAGAUUGACUUCUCGGAAU

**Table 2:** Oligonucleotides for quantitative RT-PCR.

Gene	Sequence (5′-3′)
ACTB, forward primer	GACAGGATGCAGAAGGAGATCA
ACTB, reverse primer	GCTCAGGAGGAGCAATGATCTT
MGRN1, forward primer	TGGTGAACATCCGCAAAGACT
MGRN1, reverse primer	ATCGGCGTCGAAGGTGAAC

## Data Availability

Not applicable.
